# White spot lesions, plaque accumulation and salivary caries-associated bacteria in clear aligners compared to fixed orthodontic treatment. A systematic review and meta- analysis

**DOI:** 10.1186/s12903-023-03257-8

**Published:** 2023-08-27

**Authors:** Shailaja Raghavan, Elham S. Abu Alhaija, Mandeep Singh Duggal, Srinivasan Narasimhan, Sadeq Ali Al-Maweri

**Affiliations:** 1 College of Dental Medicine, QU Health, Qatar University, Doha, P.O. Box: 2713, Qatar; 2 Hamad Dental Centre, Hamad Medical Corporation, Doha, Qatar

**Keywords:** Clear aligners, White spot lesions, Plaque index, Salivary caries-associated bacteria

## Abstract

**Objective:**

To analyse the available evidence regarding the incidence and severity of white spot lesions (WSLs), plaque accumulation and salivary caries-associated bacteria(SCB) in clear aligners (CA) verses conventional fixed (CF) orthodontic appliances.

**Methods:**

Electronic searches of MEDLINE, Scopus, Embase, Google Scholar, Clinical trial registry, OpenGrey and ProQuest were done for all relevant studies. Eligibility criteria were; Randomized Controlled Trials and Non-Randomized Studies that compared the incidence and severity of WSLs, plaque accumulation and SCB between CA and CF appliances in patients undergoing orthodontic treatment. The risk of bias(ROB) and certainty of evidence was assessed independently by two reviewers using Cochrane’s ROB and GRADEpro, respectively. Standardized mean difference (SMD) was used to estimate the effect size using STATA 17 software.

**Results:**

A total of 14 studies met the eligibility criteria, and eight were suitable for meta-analysis. The qualitative results showed lower incidence and severity of WSLs, plaque accumulation, and SCB in CA group compared to CF appliances. The pooled results showed significantly lower plaque accumulation(SMD − 1.58;95%CI:-2.57,0.58;p = 0.002) in CA compared to CF appliances.

**Conclusions:**

A moderate-quality evidence reveals less plaque accumulation and less SCB in CA, which might be related to the reduced incidence and severity of WSLs associated with CA when compared with CF appliances. However, the results of the present study should be interpreted with caution given the high ROB among some of the included studies as well as the marked heterogeneity across the studies.

**Clinical relevance:**

For patients who can be treated with either CA or CF appliances, CA may be a better choice concerning oral health.

**Registration:**

Open Science Framework (DOI:10.17605/osf.io/kcpvb).

**Supplementary Information:**

The online version contains supplementary material available at 10.1186/s12903-023-03257-8.

## Introduction

White spot lesions (WSLs) are consequences of subsurface enamel demineralization caused by acid producing caries-associated bacteria in the plaque. WSLs manifest as chalky white opacity of enamel and is an undesirable common complication of orthodontic treatment [1]. Several studies have reported a substantial increase in the prevalence of WSLs in orthodontic patients ranging from 2 to 97% [2]. Although, it is believed that these lesions may reduce or even disappear after appliance debonding due to the remineralizing potential of saliva, [3] some of the lesions may still persist much longer [4]. The significant increase in prevalence of these lesions during fixed appliance treatment is attributed to the increase in plaque retentive areas that hinder the routine oral hygiene measures, further increasing the plaque load around the brackets.

Apart from plaque accumulation, fixed orthodontic appliance induces alterations in oral microbiota; it has been reported that there are increased levels of Streptococcus mutans and Lactobacillus species in the oral cavity detected after bonding orthodontic attachments [5, 6]. Furthermore, analysis by checkerboard DNA-DNA hybridization technique has shown multi-colonization of several bacterial species including cariogenic microorganism on metallic brackets soon after bonding [7]. In addition, a recent study based on RT PCR quantification of salivary levels of caries-associated bacteria in patients with fixed orthodontic appliance revealed increased levels compared to non-orthodontic patients [8]. The increase in plaque coupled with elevation in caries-associated bacterial counts in biofilm and saliva [5] eventually reduces the pH resulting in enamel demineralization.

Recently, there has been an increase in aesthetic demands among patients seeking orthodontic treatment [9]. Clear aligners (CA) are transparent removable thermoplastic trays that is believed to be safe, aesthetic, removable and comfortable orthodontic appliance. They enable patients to carry out routine oral hygiene procedures and thereby reducing the negative effects of orthodontic appliance on periodontal health [10]. However, a 2.85% overall incidence of new WSLs has been reported with the use of CA and 28% of the patients were affected by at least one new WSL considering all the assessed teeth [11]. In addition, surface area of the WSLs has been found to be large but with less mineral loss during CA treatment compared to fixed appliance treatment [12]. This can be attributed to the fact that patients are advised to wear aligners approximately 22 h a day for optimal results which interrupts the self-cleansing activities of orofacial soft tissues allowing further accumulation of plaque under the aligner [13] and hampers the cleansing, buffering and remineralizing properties of saliva. Another study reported increase in caries- causing microbes namely, Streptococcus and Lactobacillus, within 24 h of CA wear [14].

Although, many studies on the periodontal health status, incidence of WSLs and salivary caries-associated bacterial levels in patients undergoing treatment with CA and fixed orthodontic appliances have been done, there are still some controversies existing [15]. Recent findings by Shokeen et al. [16] reported that CA treatment has less negative impact on clinical oral health outcomes than fixed orthodontic appliance. However, Chhibber et al. [17] and Pango et al. [18] reported no significant difference in the oral hygiene levels between CA and conventional brackets during long term orthodontic treatment. A study by Mummolo et al. [19] reported abundance in Streptococcus mutans during fixed appliance treatment compared to CA treatment whereas another study based on 16 S rRNA gene found no significant variations in the relative abundance of Streptococcus between the aligner and fixed appliance treatment [20].

To our best knowledge, there are no systematic reviews that have compared conventional fixed (CF) orthodontic appliance solely with CA focussing on plaque accumulation and salivary caries-associated bacteria (SCB) collectively, which have a direct influence on development and severity of WSLs. Hence, this review was conducted with the objective of systematically synthesizing all the available evidence regarding the following research question: Is there a difference in plaque accumulation measured by plaque index (PI), SCB, incidence and severity of WSLs (outcomes) in orthodontic patients (population) undergoing CA (intervention) and CF orthodontic appliance (control) treatment?

## Materials and methods

### Protocol and registration

This review was conducted in accordance with Cochrane Handbook for Systematic Reviews of Interventions and the Preferred Reporting Items for Systematic Reviews and Meta-Analyses (PRISMA) 2020 statement [21]. The protocol of the systematic review was registered in OPEN SCIENCE FRAMEWORK registries (DOI: 10.17605/osf.io/kcpvb).

### Eligibility criteria

The eligibility criteria applied for the systematic review are presented in Table 1.

**Table 1:** The eligibility criteria applied for the systematic review and Meta- analysis

	Inclusion criteria	Exclusion criteria
Population	-Patients with normal general health -Patients requiring comprehensive orthodontic treatment -Dentition: Late mixed and permanent dentition -No active caries lesion	-Pre-existing periodontal or salivary gland diseases -Patients under antibiotics or analgesics
Intervention	-Clear aligners	
Control	-Conventional fixed orthodontic appliance	-Self- ligating brackets -Lingual appliance
Outcome	-Incidence of WSLs -Severity (Depth and Surface area of lesion) of WSLs -Plaque index -Salivary cariogenic bacteria (Streptococcus mutans and Lactobacillus acidophilus)	
Study design	-RCTs -Cohort studies	-Cross sectional studies -Case-control studies -Case reports -Case series
Language	-English	

### Information sources, search strategy and study selection

A 3-month comprehensive electronic database search was conducted from January 2022 up to May 2023. Literature (1990- May 2023) from relevant databases: PubMed, Scopus and Embase, and Google Scholar were included in the review. Search of the grey literature was also performed on Opengrey and Proquest Dissertation Abstracts and Thesis database. An additional search was performed in ClinicalTrials.gov (www.clinicaltrials.gov). A combination of index terms (Medical Subject Headings (MeSH) for PubMed and other relative terms pertaining to the databases) and keywords were used to perform search. The detailed search strings are presented in the Table 2. Hand searching was performed from the reference lists of included articles and published systematic reviews.

**Table 2:** The search strategy used in the review using MeSH terms (PubMed), key words and terms related to other database

S. No	Database	Search strategy
1	Medline via PubMed	
	WSLs	((((orthodontic appliance, removable) OR (clear aligner)) OR (invisalign)) OR (thermoplastic orthodontic appliance)) AND (((((((((Dental caries) OR (Deminerali*)) OR (White spot lesion)) OR (Incipient lesion)) OR (Incipient carious lesion)) OR (Early enamel caries)) OR (Early enamel carious lesion)) OR (Subsurface enamel lesion)) OR (Subsurface lesion))
	Plaque index	(((orthodontic appliance, removable) OR (clear aligner)) OR (invisalign)) OR (thermoplastic orthodontic appliance)) AND (((((Periodont*) OR (Oral health)) OR (Oral hygiene)) OR (Plaque index)) OR (PI))
	Salivary caries-associated bacteria	((((orthodontic appliance, removable) OR (clear aligner)) OR (invisalign)) OR (thermoplastic orthodontic appliance)) AND (((((microb*) OR (microorganism)) OR (streptococc*)) OR (lactobacill*)) OR (microflora))
2	Scopus	
	WSLs	(aligner OR thermoplastic aligner OR clear aligner OR removable orthodontic appliance OR invisalign AND tooth demineralization OR white spot lesion OR early enamel caries OR subsurface lesion OR incipient caries OR dental caries)
	Plaque index	(aligner OR thermoplastic aligner OR clear aligner OR removable orthodontic appliance OR invisalign AND oral hygiene OR oral health OR periodontal health OR dental plaque index OR PI OR plaque)
	Salivary caries-associated bacteria	(aligner OR thermoplastic aligner OR clear aligner OR removable orthodontic appliance OR invisalign AND microorganisms OR microbiome OR microbes OR microflora OR lactobacillus OR streptococcus OR lactobacilli OR streptococci)
3	Embase	
	WSLs	(aligner OR ‘orthodontic aligner’ OR (thermoplastic AND clear AND aligner) OR ‘removable orthodontic appliance’ OR ‘invisalign’) AND (‘white spot lesion’ OR ‘dental caries’ OR (tooth AND demineralization) OR (enamel AND demineralization) OR ‘demineralization’ OR ‘decalcification’ OR (incipient AND lesions) OR (subsurface AND lesion))
	Plaque index	(aligner OR ‘orthodontic aligner’ OR (thermoplastic AND clear AND aligner) OR ‘removable orthodontic appliance’ OR ‘invisalign’) AND (‘periodontal health’ OR ‘oral health’ OR ‘oral hygiene’ OR ‘plaque index’ OR ‘oral health status’
	Salivary caries-associated bacteria	(aligner OR ‘orthodontic aligner’ OR (thermoplastic AND clear AND aligner) OR ‘removable orthodontic appliance’ OR ‘invisalign’) AND (‘microorganism’ OR ‘microflora’ OR ‘streptococcus’ OR ‘lactobacillus’ OR ‘microbiome’)
4	Google scholars	
	WSLs and Plaque index and Salivary caries-associated bacteria	https://scholar.google.com/scholar?
5	ProQuest and Opengray	
	WSLs and Plaque index and Salivary caries-associated bacteria	clear aligners AND whitespot lesions OR caries AND plaque index AND salivary cariogenic bacteria OR salivary microbiome

All the identified records were imported into reference management software (desktop version of EndNote®, version X9; Clarivate Analytics). After removal of duplicates, two reviewers (S.R and E.A) independently screened the articles based on title and abstracts using Rayyan Systematic Review Screening Software (https://www.rayyan.ai). The full texts of potentially eligible studies and those with insufficient information in the abstract were retrieved and read in full for the final selection. Articles that did not meet any one or more of the inclusion criteria were excluded. Any disagreements in screening and including potentially relevant articles were resolved by a third reviewer (S.A).

### Data extraction

Data extraction was performed by two reviewers (S.R and E.A) independently using a standardized data extraction form that comprised the following items:

- Study information: Author, year of publication, study design, study setting and sample size and funding.
- Population: Age, gender.
- Intervention and control: Type of appliance.
- Outcomes: Outcome measures (pertaining to the review), method of obtaining the outcome measures, follow-up periods and results . For few studies [16, 22] Web plot digitizer (https://automeris.io/WebPlotDigitizer/website) was used to extract the data from the graphs and plots. Some of the authors were contacted through email for obtaining clarifications, missing and additional data.

### Risk of bias (ROB) assessment in individual studies

A revised version of Cochrane risk-of-bias tool ROB 2 [23] and ROBINS-I [24] was used to assess the risk of bias in Randomised Control Trials (RCTs) and non-randomized studies of intervention (NRSIs), respectively. The risk of bias assessment tool for RCTs is based on the following 5 domains to evaluate the risk of bias as a result of: randomization process, deviation from intended intervention, missing outcome, inappropriate measurement of outcome and selective reporting of results. The overall judgment can be of ‘low/ high risk of bias’ or can express ‘some concerns. The risk of bias assessment tool for NRSIs is based on the following 7 domains to evaluate the risk of bias due to confounding, selection of participants, classification of interventions, deviation from intended intervention, missing outcome, inappropriate measurement of outcome and selection of reported results. The overall judgment can be of ‘low/ moderate/ serious/ critical risk of bias or ‘no information’. All the extracted data were cross-verified by 2 reviewers (S.R and E.A) any discrepancies were resolved by the third reviewer (N.S).

### Summary measures and synthesis of results

For the effect size calculation, the mean and standard deviation (SD) were extracted from all the included studies. In the absence of means and SDs, these were derived from the reported medians, inter-quartile ranges, or confidence intervals (C.I.s). As the eligible studies assessed the PI at varying and multiple time-points, the data for the maximum time-point from each of the included studies was considered for meta-analysis. The influence of the time-point on the effect size was considered as continuous moderator (assessment duration in months) in the meta-regression analysis. Sub-group analysis was conducted for categorical moderator based on the study-design (RCT and NRSI). In addition, three separate meta-analysis were conducted based on the PI follow-up duration (at 3, 6 and 12 months) to assess effect size at these time-points.

The standardized mean differences (SMD) and 95% C.I. for PI was used in the summary measures. Random effects meta-analysis with restricted maximum likelihood method was conducted. Statistical heterogeneity was first examined through visual inspection of the C.I. for the treatment effects on forest plots. A chi-square (*p*-value below the level of 10%) was considered as indicative of significant heterogeneity) and *I*^2^ tests (value greater than 50% was considered as substantial heterogeneity) were applied to assess the heterogeneity. Predictive intervals (95% P.I.) were calculated to incorporate existing heterogeneity and to provide a range of possible effects in future studies. All the analyses were performed using STATA 17 software (StataCorp, College Station, TX).

### Risk of bias across the studies

Contour- enhanced funnel plot was decided to be generated to assess publication bias if at least 10 studies were to be included in the meta-analysis.

### Quality of evidence

The certainty of evidence using the GRADE approach was used to rate the quality of evidence of estimates (high, moderate, low, and very low) derived from the MA using GRADEpro GDT software (https://www.gradepro.org). The GRADE summary of findings was categorized based on the study design. Accordingly, two tables were created for the plaque accumulation (RCT and NRSI). Two reviewers (S.R and N.S) independently assessed the confidence in effect estimates for outcomes synthetized quantitatively using the following categories: risk of bias, inconsistency, indirectness, imprecision, and publication bias.

## Results

### Study selection and characteristics

**Fig. 1:**
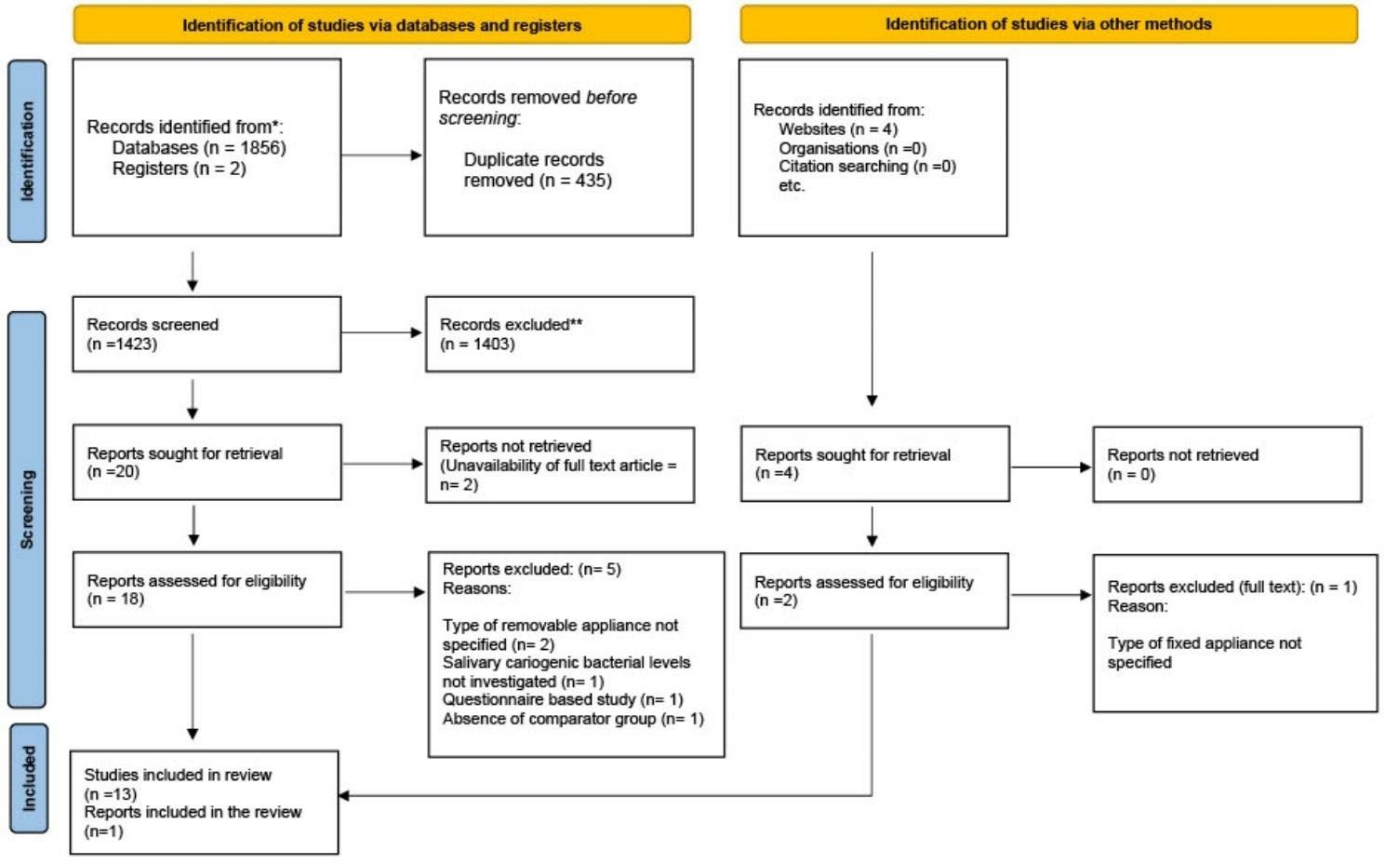
The PRISMA 2020 Flow Diagram of article retrieval

The results of search and study selection are shown in Fig. 1. The total number of reports identified was 1862 (1858 from the databases and registry and 4 reports from grey literature). After removal of 435 duplicates, 1427 reports were included in the title and abstract screening. Of these, 1403 from the databases and 2 from grey literature were excluded and only 22 reports (20 from the databases and 2 grey literature) were entitled for full text screening. Due to non-availability of 2 full text reports from databases, 20 reports (18 from the databases and 2 grey literature) were screened. The full texts of potentially eligible articles were assessed by the two reviewers (S.R and E.A), of which 6 (5 from the databases and 1 grey literature) were excluded for various reasons. The list of excluded studies along with reason of exclusion is summarized in online resource 1. Finally, 14 articles [12, 16–19, 22, 25–32] were included for qualitative analysis out of which 8 articles were suitable for meta-analysis.

The characteristics of included studies are depicted in Tables 3, 4 and 5. This systematic review included 5 RCTs [12, 17, 26, 27, 31] and 9 NRSI [16, 18, 19, 22, 25, 28–30, 32]. Four of the included studies [12, 29–31] investigated the incidence of WSLs, eight studies [16–18, 22, 26–28, 32] investigated plaque accumulation as one of the primary outcomes and two studies [19, 25] investigated SCB as primary outcome and plaque accumulation as additional outcome.

**Table 3:** Characteristics of included studies reporting incidence and severity of WSLs comparing CAs and CF orthodontic appliances

Author, Year	Study design and study setting	Sample size M\F	Age (years)	Method of evaluation	Result for all outcomes			Funding
Buschang et al., 2019 [29]	Retrospective Cohort Private practice and at the Department of Orthodontics, Texas A&M University College of Dentistry.	CA-244 (64% F) CF- 206 (63% F)	CA: 30.4 ± 14 CF: 29.2 ± 11.5	Digital photographs	Percentage of patients developing new lesions in the maxillary teeth (at the end of orthodontic treatment)	CA	0.8%	Not mentioned
						CF	18.9%	
					Percentage of patients developing new lesions in the Mandibular teeth	CA	0.4%	
						CF	15.3%	
Albhaisi et al., 2020 [12]	RCT Postgraduate orthodontic clinic at Jordan University of Science and Technology.	CA-27 (7 M/20F) CF- 22 (3 M/19F)	CA- 21.2 CF- 21.3	QLF	Number of newly developed lesions	CA	143	Not mentioned
						CF	165	
					Avg. fluorescence loss ( T1- T0)	CA	-0.4	
						CF	-1.2	
					Lesion area in pixels ( T1- T0)	CA	82.2	
						CF	9.3	
Dallel et al., 2020 [30]	Prospective cohort CFculty of Dental Medicine, University of Monastir, Monastir, Tunisia	CA- 47 (25 M/22F) CF- 31 (14 M/17F)	CA- 15.6 ± 3 CF-16.75 ± 2	Visual examination	Percentage of newly developed lesions	CA	7	Not mentioned
						CF	29	
Hussin Alshatti, 2017 [31]	RCT Division of Orthodontics, Department of CranioCFcial Sciences, at the University of Connecticut Health Center	CA- 15 CF- 15 Gender distribution not mentioned	CA- 21.44 ± 11.63 CF- 14.47 ± 3.99	Digital photographs	Percentage of patients developing new lesions ( From T0 to T2)	CA	41.18	Not mentioned
						CF	52.94	
					Lesion surface area (Mean T2- Mean T0)	CA	0.06 (0.09)	
						CF	0.09 (0.11)	

**Table 4:** Characteristics of included studies reporting Plaque accumulation using Plaque Index (PI) comparing CAs and CF orthodontic appliances

Author, Year	Study design and study setting	Sample size M\F	Age (years)	Outcome measures (Index used)	Results at different follow-up time period; Mean (SD)			Funding
Abbate et al., 2015 [26]	RCT Department of Orthodontics, School of Dentistry, University of Insubria, Varese, Italy	CA-25 CF- 25 Gender distribution not mentioned	10–18	Mean plaque index score (Plaque index of Silness and Loe)	3 M^*^	CA	0.64 (0.48)	Not mentioned
						CF	1.92 (0.63)	
					6 M	CA	0.31 (0.47)	
						CF	2.32 (0.65)	
					12 M	CA CF	0.36 (0.48) 2.42 (O.6)	
Chhibber et al., 2018 [17]	RCT The outpatient orthodontic clinic, University of Connecticut Health, Farmington, Conn	CA-27 (20 M/7F) CF − 22 (12 M/10F)	16.56 ± 3.99 14.56 ± 3.92	Mean plaque index score (Modified index of Loe)	9 M	CA	0.83 (0.48)	Align Technology, San Jose, California
						CF	1.32 (0.67)	
					18 M	CA	0.92 (0.58)	
						CF	1.32 (0.67)	
Karkhanechi et al., 2013 [22]	Prospective cohort Department of Orthodontics, College of Dentistry, New York University	CA- 20 (8 M/12F) CF- 22 (6 M/16F)	28 ± 6.86 34 ± 7.18	Mean plaque index score (Modified index of Loe)	1.5 M	CA	0.67 (0.44)	Not mentioned
						CF	0.88 (0.45)	
					6 M	CA	0.67 (0.38)	
						CF	1.26 (0.38)	
					12 M	CA	0.62 (0.29)	
						CF	1.13 (0.29)	
Levrini et al., 2013 [27]	RCT Department of Orthodontics, School of Dentistry, University of Insubria, Italy	CA- 10 (3 M/7F) CF- 10 (3 M/7F)	24.6 ± 6.4 25.7 ± 3.4	Mean plaque index score (Modified index of Loe)	1 M	CA	0.35 (0.48)	Not mentioned
						CF	0.95 (0.94)	
					3 M	CA	0.4 (0.59)	
						CF	1.15 (0.67)	
Miethke et al., 2005 [32]	Concomitant trial Department of Orthodontics and Dentofacial Orthopedics of the Charité Berlin.	CA- 30 CF30 (43 M/17F)	18–51 (30.1)	Mean plaque index score (Modified index of Loe)	6 M	CA	0.48 (0.41)	Not mentioned
						CF	0.8 (0.58)	
					7 M	CA	0.41 (0.37)	
						CF	0.56 (0.44)	
					8 M	CA	0.28 (0.32)	
						CF	0.5 (0.53)	
Mummolo et al., 2020 April [19]	Prospective cohort A dental clinic at Abruzzo (Central Italy)	CA-40 (24/ 16) CF-40 (22/18)	20.4 ± 1.7 21.3 ± 1.7	Mean plaque index score (Index not specified)	3 M	CA	0 (0.0000)	Not mentioned
						CF	0.7 (0.55)	
					6 M	CA	0 (0.0000)	
						CF	1.4 (0.5)	
Mummolo et al., 2020 [25]	Prospective cohort A dental at Abruzzo (Central Italy)	CA-30 CF- 30	21.5 ± 1.5 23.3 ± 1.6	Mean plaque index score (Index not specified)	3 M	CA	0 (0.0000)	Not funded
						CF	0.7 (0.59)	
					6 M	CA	0 (0.0000)	
						CF	1.3 (0.46)	
Pango et al., 2020 [18]	Prospective cohort Section of Orthodontics and Temporomandibular Disorders of the University of Naples Federico II, Italy	CA- 20 (5/15) CF- 20 (9/11)	34.7 ± 12.5 20.6 ± 8.1	Median IQR (Plaque index by Silness and Loe)	3 M	CA	11.93^a^ (16.75)	Not funded
						CF	13.96^a^ (16.35)	
Shokeen et al., 2022 [16]	Prospective cohort University of California Los Angeles (UCLA) School of Dentistry, Orthodontics Clinic	CA- 12 (4/8) CF- 12 (4/8)	29 ± 12 22 ± 13	Mean plaque index score (Turesky Modified Quigley Hein Plaque Index)	1 M	CA	1.31 (1.43)	University of Buffalo and The Forsyth Institute
						CF	2.07 (1.91)	
					3 M	CA	2.04 (0.90)	
						CF	2.17 (0.97)	
					6 M	CA	1.70 (0.90)	
						CF	2.45 (0.97)	
					12 M	CA	1.71 (0.83)	
						CF	2.64 (0.79)	
Levrini et al., 2015 [28]	Prospective cohort Department of Orthodontics of the University of Insubria.	CA- 32 (5/27) CF- 35 (18/17)	16–30 (24.3)	Mean plaque index score (Modified index of Loe and Silness)	No data available			Not funded

^*^ M: Months ^a^ Median (IQR) converted to Mean

**Table 5:** Characteristics of included studies reporting Salivary Cariogenic Bacteria (SCB) comparing CAs and CF orthodontic appliances (Included for quantitative synthesis)

Author, Year	Study design and study setting	Sample size M\F	Age (Years)	Outcome measures	Results at different follow-up time period				Funding
Mummulo et al., 2020 [19]	Prospective cohort A dental clinic at Abruzzo (Central Italy)	CA-40 (24 M/ 16 F) CF-40 (22 M/18F)	20.4 ± 1.7 21.3 ± 1.7	Number of subjects with S. mutans and Lactobacilli CFU/ml > 10^5^	3 M	SM^a^	CA	0	Not mentioned
							CF	8	
						LB^b^	CA	0	
							CF	8	
					6 M	SM	CA	3	
							CF	15	
						LB	CA	1	
							CF	15	
Mummulo et al., 2020 [25]	Prospective cohort A dental clinic at Abruzzo (Central Italy)	CA- 30 (18 M/12F) CF- 30* (22 M/12F) *	21.5 ± 1.5 23.3 ± 1.6	Number of subjects with S. mutans and Lactobacilli CFU/ml > 10^5^	3 M	SM	CA	0	Not funded
							CF	6	
						LB	CA	1	
							CF	8	
					6 M	SM	CA	3	
							CF	12	
						LB	CA	4	
							CF	12	

^a^SM = Streptococcus mutans ^b^LB = Lactobacilli *As given in the article

Out of 4 studies that assessed the WSLs, two were RCTs [12, 31] and two were NRSIs [29, 30]; one study followed a retrospective design [29] and the other a prospective design [30]. The method used to assess the incidence were different across the study. Two studies [29, 31] used digital photographs, 1 study used Quantitative Light Fluorescence [12] and 1 study assessed WSLs by visual examination [30]. Among these, only 2 studies [12, 31] assessed the severity of WSLs in terms of surface area and 1 study [12] in terms of the depth of lesions.

Out of the 10 studies that evaluated plaque accumulation (as primary or additional outcome) using PI, three were RCTs [17, 26, 27] and the others [16, 18, 19, 22, 25, 28, 32] were observational studies. The indices used to measure the outcome and the time points of plaque quantification were varying across the studies. Two studies [18, 26] used PI of Silness and Loe, five studies [17, 22, 27, 28] used Modified plaque index of Loe, one study [16] used Turesky Modified Quigley Hein Plaque Index and two studies [19, 25] did not mention the index used. The two observational studies [19, 25] that investigated salivary caries-associated bacteria had assessed the number of subjects (percentage) with Streptococcus mutans and Lactobacilli greater than 10^5^ CFU/ml as outcome measure. All the outcomes were measured at different time points ranging from 1 to 18 months.

### Risk of bias within studies

All the included studies had limitations in methodology that contributed to bias. The overall risk of bias of all included RCTs [12, 17, 26, 27, 31] and three NRSIs [22, 29, 32] were graded as high risk whereas the risk of bias of the other five NRSIs [16, 18, 19, 25, 30] were graded as moderate. Risk of bias of one of the studies [28] could not be judged as there was no information on the missing data.

-RCTs.

**Fig. 2:**
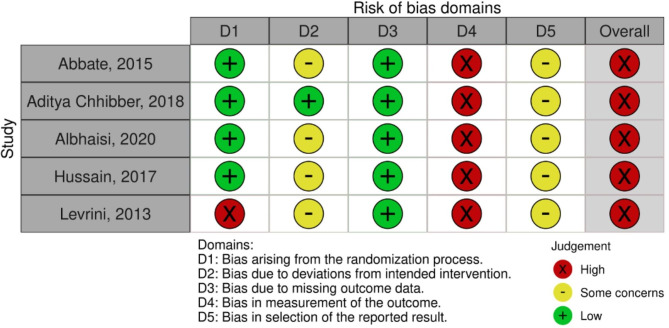
Risk of Bias summary outlining judgement of ROB items of Randomized controlled trials using – ROB2

The risk of bias assessment and the overall judgement is shown in Fig. 2. All included RCTs [12, 17, 26, 27, 31] suffered high risk of bias in measurement of the outcome mainly due to lack of blinding of assessors and only one study [27] was additionally graded high risk of bias arising from the randomization process. Four studies [12, 26, 27, 31] showed some concerns in the bias due to deviations from intended intervention and all studies in the bias in selection of the reported result. All studies were at low risk of bias due to missing outcome data.

### -NRSI

**Fig. 3:**
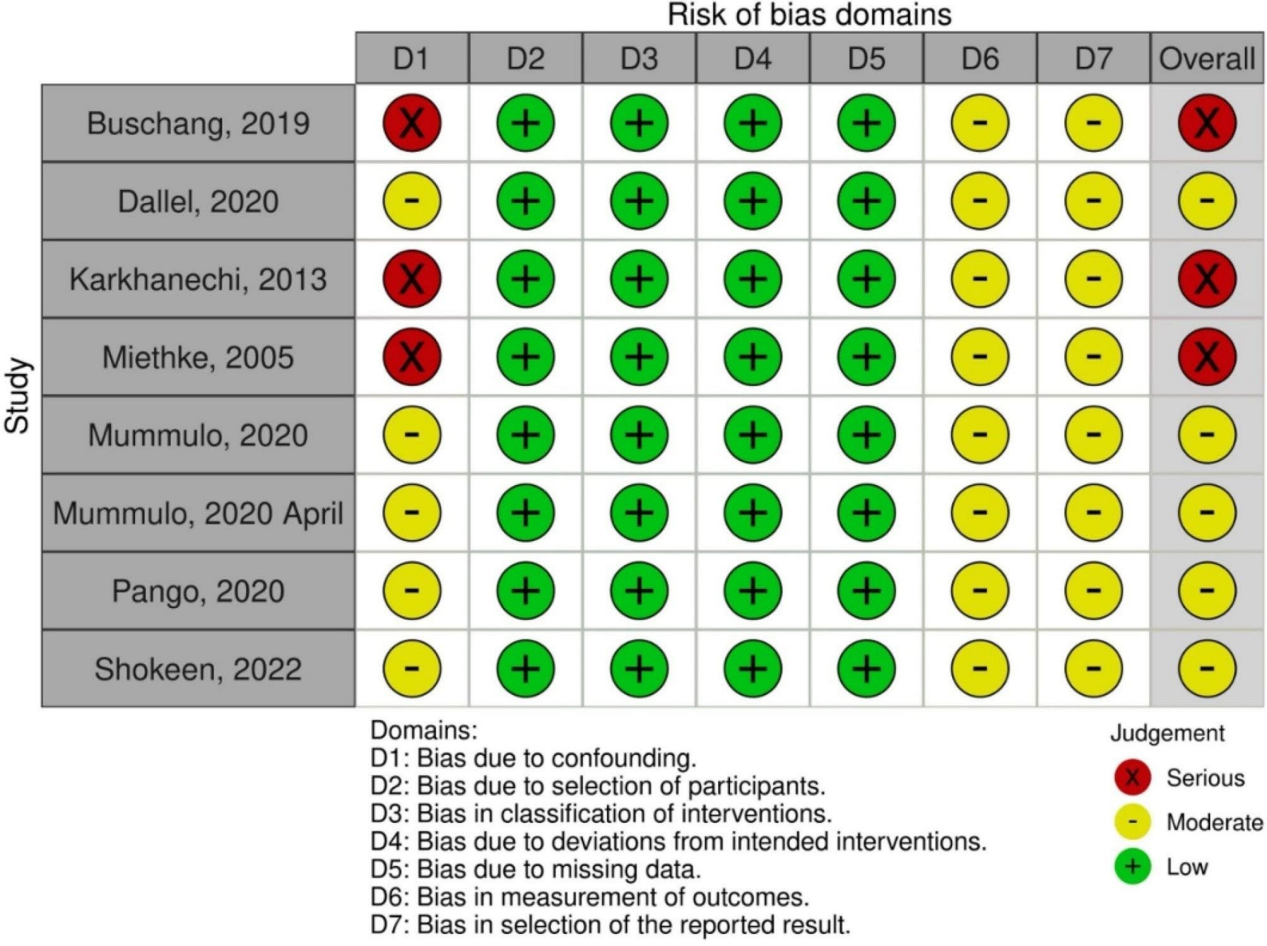
Risk of Bias summary outlining judgement of ROB items of Non randomised studies of Interventions using ROBINS- I

The risk of bias assessment and the overall judgement is shown in Fig. 3. Three included NRSI [22, 29, 32] were graded as high risk of bias and other five studies [16, 18, 19, 25, 30] were graded as moderate risk due to confounding factors. All studies suffered moderate risk of bias in domains 6–7. On the other hand, all studies were at low risk of bias in domains 2–5.

## Results of individual studies and meta-analysis

- **Plaque accumulation**.

**Fig. 4:**
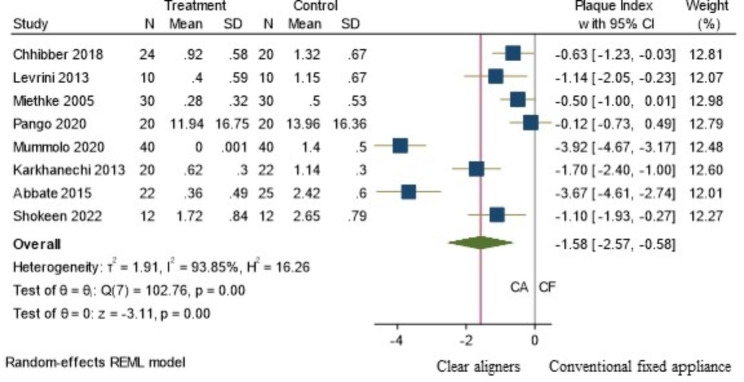
Meta- analysis (Random effects model): Forest plot comparing PI in patients with CA (Treatment) to those with CF orthodontic appliances (Control) (N-No. of Samples, SD: Standard deviation, CI: Confidence interval)

Among the 10 included studies [16–19, 22, 25–28, 32] less plaque accumulation and better oral hygiene maintenance was reported by eight of them [16, 19, 22, 25–28, 32] and only two [17, 18] found no difference between the two groups. Only eight studies [16–19, 22, 26, 27, 32] were included in the meta-analysis out of which 3 were RCTs and 5 were NRSIs. The observed SMD ranged from − 3.92 to -0.12, all the estimates being negative (100%) favouring the CA. The estimated average SMD based on the random-effects model was − 1.58 (95% CI: -2.57 to -0.58) and it differed significantly (z = -3.11, p = 0.002) favouring lesser plaque accumulation in the CA as depicted in the forest plot (Fig. 4).

**Fig. 5:**
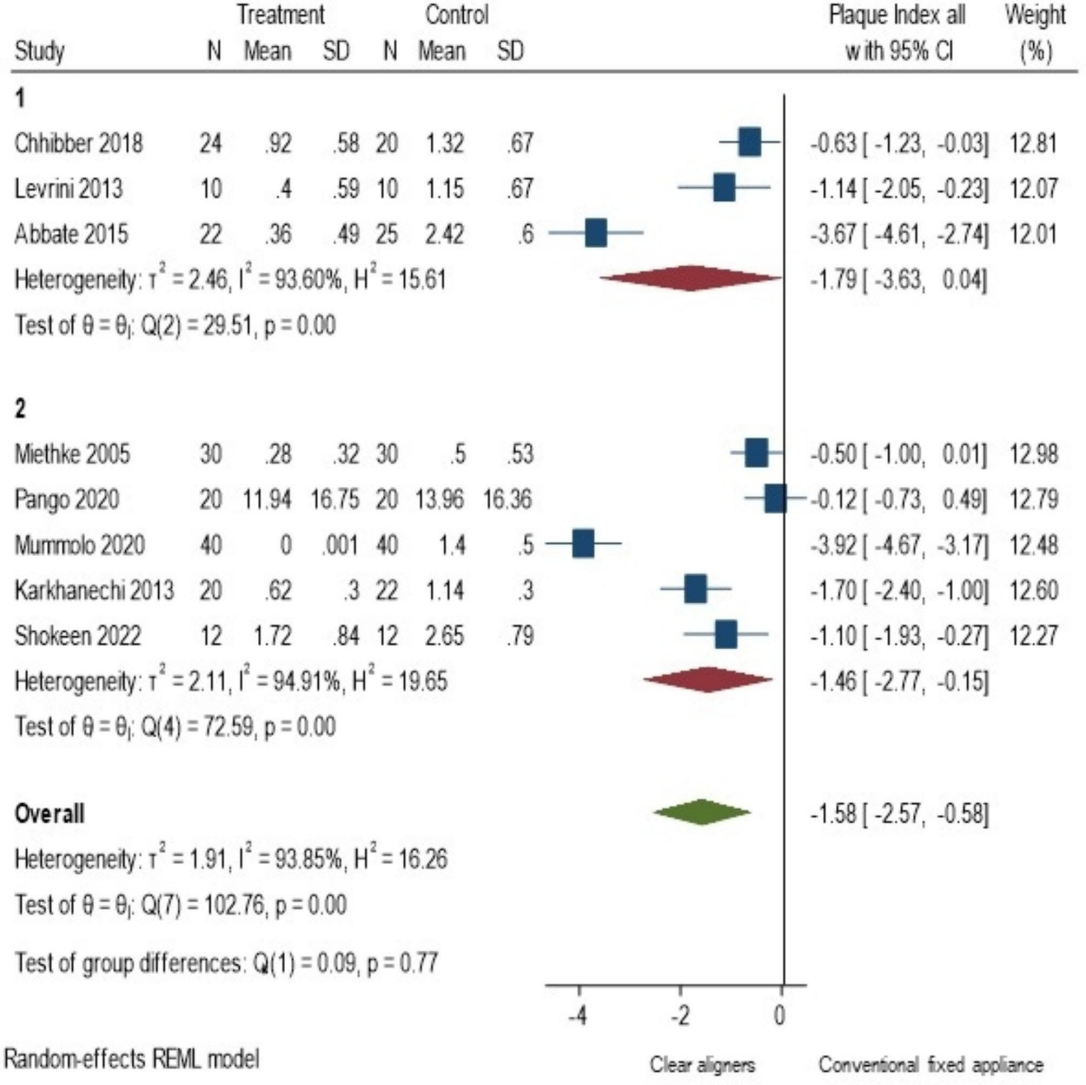
Subgroup analysis of studies reporting plaque accumulation based on study design CA (Treatment); CF orthodontic appliances (Control) (N-No. of Samples, SD: Standard deviation, CI: Confidence interval) (RCT: Randomized Controlled Trial, NRSI –Non Randomised Studies of Interventions)

According to the Q-test, the true outcomes appear to be heterogeneous (Q (7) = 102.76, p < 0.0001, tau² = 1.91, I² = 93.85%). Subgroup analysis (Fig. 5), based on the type of study design revealed the same trend favouring CA and the heterogeneity was found to be high in both the subgroups (RCT − 1.79, 95% CI: [-3.629, 0.043] and I² = 93.6%, NRSI − 1.457 95% C.I.: -2.766, -0.147] and I² = 94.91%). Meta regression analysis using duration as continuous moderator reported I^2^ residual statistic as 94.57%, which still suggests high heterogeneity (online resource 3). In addition, duration did not influence the effect size (z=-0.09, p = 0.93). A 95% P.I. (online resource 2) was found to be -5.181 to 2.025 which indicates that the possibilities of the estimate to be positive in the future studies, though the average outcome is estimated to be negative.

**Fig. 6:**
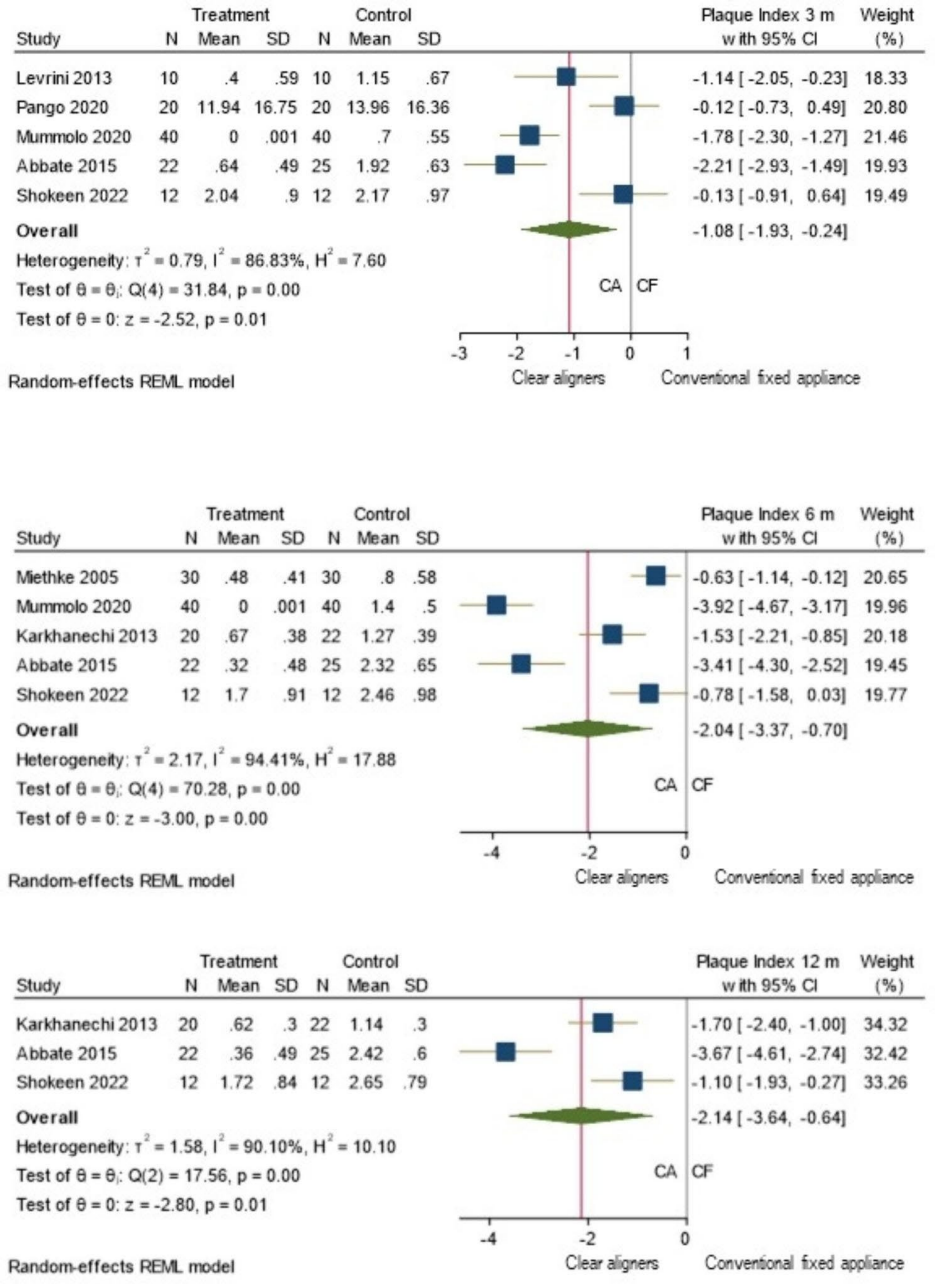
a-c: Separate Meta- analysis (Random effects model): Forest plot comparing PI in patients with CA (Treatment) to those with CF orthodontic appliances (Control) at 3 months, 6 months and 12 months (N-No. of Samples, SD: Standard deviation, CI: Confidence interval)

Three separate forest plots showing the pooled effect size with 95%C.I. for the time-points 3, 6 and 12 months were presented in Fig. 6a-c. The number of studies with 3 months and 6 months was five, and with 12 months follow up was three.

At all the three time points, the effect size was favouring CA and it is statistically significant (P < 0.05).

b. **Salivary caries-associated bacteria**.

Both the included studies reported higher concentration of caries-associated bacteria in CF orthodontic appliances as compared to CA [19, 25]. Meta-analysis of the SCB outcome was not carried out due to lack of sufficient number of studies.

c. **White spot lesions**.

Only four studies [12, 29–31] that assessed the incidence of WSLs were available. Less risk of developing WSLs in CA than CF orthodontic appliances was reported by three studies [12, 29, 30] whereas one study reported no difference in the incidence and severity of WSLs between CA and CF orthodontic appliances [31]. As different methodologies were adopted in each one of the studies, meta-analysis for incidence and severity of WSLs was not possible.

### Risk of bias across studies

Due to sparse datasets included in the synthesis that assessed PI, funnel plots were not generated to assess publication bias.

### Quality of evidence

The GRADE summary of findings (strength of evidence for interventions) substantiated the evidence for less plaque accumulation in the clear aligner patients (Table 6).

**Table 6:** Certainty of evidence - Based on the Grading of Recommendations Assessment, Development and Evaluation Approach – (GradePro GDT)

Certainty assessment							№ of patients		Effect		Certainty	Importance
№ of studies	Study design	Risk of bias	Inconsistency	Indirectness	Imprecision	Other considerations	Clear aligner	Fixed appliance	Relative (95% CI)	Absolute (95% CI)		
Plaque accumulation - Randomized trials (follow-up: range 1 months to 18 months; assessed with: Plaque Index)												
3	randomised trials	serious^a^	serious^b^	not serious	not serious	strong association	56	55	-	SMD **1.79 SD lower** (3.63 lower to 0.04 higher)	⨁⨁⨁◯ Moderate	IMPORTANT
**Plaque accumulation - NRSI (follow-up: range 1 months to 12 months; assessed with: Plaque Index)**												
5	observational studies	serious^c^	serious^d^	not serious	not serious	strong association	122	124	-	SMD **1.46 SD lower** (2.77 lower to 0.15 lower)	⨁⨁⨁◯ Moderate	IMPORTANT

**CI**: Confidence interval; **SMD**: Standardised mean difference

In context with the evidence from I^2^ statistics for heterogeneity and ROB 2/ROBINS-I tool for risk of bias, downgrading for inconsistency and Risk of Bias domains for plaque accumulation was implemented. On the other hand, the evidence rating was upgraded for strong association as the quantitative pooling of the results showed a large effect. The results revealed that the quality of evidence for the plaque accumulation was graded as “moderate” for both RCT’s and NRSIs.

## Discussion

Adult orthodontic patients tend to prefer CA over CF appliances as it satisfies their aesthetic demands and is proven to have a positive impact on the QoL [9]. Generally, quality of life (QoL) has been reported to reduce during orthodontic treatment and the type of orthodontic appliance is said to influence the patients functionally and psychologically [33]. However, it has been shown that CAs cause less physical and psychological disabilities compared to fixed appliances [33]. Based on existing literature, it is believed that an increase in the quantum of plaque, caries-associated bacteria in saliva, reduction in the salivary pH and resultant enamel demineralization are unwanted sequel of orthodontic treatment jeopardising the aesthetics offered by orthodontic treatment. There are individual studies comparing the plaque accumulation, SCB levels, and the incidence and severity of WSLs in patients undergoing treatment with CA to that of CF orthodontic appliances [12, 16, 17, 19, 29]. One review was identified that compared clear aligners with fixed orthodontic appliance in terms of the 3 variables (WSLs, PI and SCB) [34]. The aforementioned study lacked robust eligibility criteria; intervention group included any orthodontic treatment with aligners and comparator group included fixed orthodontic treatment, other aligner treatment or removable appliances. The comparator group in the review not only included conventional fixed appliance but also Self- ligating and lingual appliances, which, among themselves, exhibit difference in quantum of plaque accumulation and SCB due to bracket design and placement. The above mentioned differences among the types of fixed appliance reflect on the incidence of WSLs which would also vary. Hence, this review was conducted to synthesize explicit evidence of any possible link between the incidence and severity of WSLs, plaque accumulation and SCB in an attempt to distinguish these parameters between CA and CF.

Studies that compared the CA with CF orthodontic appliances were only included. Other types of fixed orthodontic appliance such as self-ligating or lingual appliances were excluded due to the controversies in the literature related to the influence of bracket type on WSLs, the quantity of plaque accumulation and cariogenic microbial colonization [35–42]. PI and SCB levels (Streptococcus mutans, in particular) were considered appropriate outcome variables as they are proven to be the best predictors of WSLs [43].

The qualitative assessment of the included studies indicated comparatively lower incidence and severity of WSLs and SCB with CA as opposed to CF orthodontic appliances which could be attributed to fewer plaque retention sites and ease of oral hygiene maintenance with CAs. In addition, plaque accumulation as assessed with PI was less in CA than CF orthodontic appliances. These findings are consistent with those of previous reviews [15, 34, 44]. However, the aforementioned reviews included self-ligating and lingual appliances in addition to CF orthodontic appliances, and their inclusion was regarded as a reason for heterogeneity. Also, no attempts were made to explore heterogeneity in one of the systematic review while many potential articles missed their way into the meta- analysis (only 4 studies were included) even though the search was run until May 2021 [34].

Due to the large variability in the methods of WSLs evaluation and recording criteria (tooth-based incidence [12, 30] and patient-based incidence [29, 31]) adopted in the included studies and scarcity of studies that assessed SCB, meta-analysis for both of the mentioned outcomes was not feasible. However, quantitative analysis was conducted including studies that assessed plaque accumulation. Only eight of ten studies with PI as outcome measure was considered for the analysis. Due to lack of data, one of the study was not included into the analysis [28] and out of the two studies [19, 25] that assessed for PI as secondary outcomes, only one with higher sample size [19] was included for quantitative analysis due to the possibility of study population being mutually inclusive as both the studies were conducted in similar setting and published in the same year by the same authors. Plaque accumulation in the included studies was assessed using different plaque indices and different teeth type and number. Therefore, it was considered prudent to adopt SMD as summary measure for PI instead of mean difference used in the previous review [44]. The findings of this review indicated that CA was associated with less plaque accumulation, less salivary caries-associated bacteria and reduced incidence and severity of white spot lesions than CF orthodontic appliances.

The primary meta-analysis was conducted by including all the possible studies irrespective of variation in the duration of PI assessment and the influence of duration was considered as continuous covariate in meta regression model, which allowed us to pool maximum studies thereby increasing the power of meta-analysis. It is important to emphasize that only one dataset (dataset for maximum time-point) was taken from each of the included study to avoid the influence of dependency of data, which was not considered in the previous review [44]. Our meta-regression analysis revealed no relationship between plaque accumulation and follow-up duration. In addition, separate meta-analysis based on 3 time points (3,6 and 12 months) did not change the direction of the results. Furthermore, subgroup analysis based on study design did not identify the source of heterogeneity between RCT and NRSI. The 95% prediction interval (ranged from − 3.89 to 1.05) revealed that although the average value was more towards the direction favouring CA, there is a possibility of absence of effect or that the true effect may be in the opposite direction.

It is crucial to emphasize that this review was majorly based on studies with high risk of bias assessment. Lack of blinding of assessors (not possible due to the nature of interventions), failure to incorporate a random element in generating the allocation sequence, selective reporting, lack of consideration of confounding factors (age, gender, type of malocclusion, oral hygiene status) or failing to adjust for the confounding factors were among the reasons for this assessment. As all the included studies were of high risk of bias, stratified analysis based on ROB was not possible. Therefore, all available data were included in the meta-analyses as suggested by the Cochrane Handbook [45].

Assessment of publication bias was not feasible due to scarcity of studies that assessed plaque accumulation. Only one study [31] out of 4 reports was included from the grey literature. It is important to note that although this number is limited it adds to the strength of this review. It indicates that the possibility of missing any relevant studies is a minimum and the number of papers published in a non-indexed journals is limited.

### Limitations

The study level limitations include: high risk of bias among the included studies, and clinical and/or methodological heterogeneity across the studies that affected the certainty and generalizability of evidence. Furthermore, inability to access all eligible studies (non- English and non-availability of full texts) and scarcity of primary studies that investigated the WSLs and SCB could be regarded as limitation at review level.

## Conclusion

- Based on moderate quality evidence, CA is associated with less plaque accumulation than CF orthodontic appliances. In addition, salivary caries-associated bacteria were found to be less with CA which may be related to the reduced incidence and severity of WSLs in CA as opposed to CF orthodontic appliances.
- Future considerations should be aimed at conducting a high-quality RCT to detect the direct association of WSLs, plaque accumulation and SCB following standardized protocols in terms of study design (randomization), selection of subjects, method of evaluation of WSLs and SCB (quantitative, proper time-points of follow up measurements). Also, to ensure generalizability of the results a multi-centre study will be preferable. Additionally, RCTs employing pre-post design (before commencement and immediately after completion of orthodontic treatment) would minimize the risk of bias due to lack of blinding.

### Electronic supplementary material

Below is the link to the electronic supplementary material.

Supplementary Material 1

## Data Availability

The datasets used and/or analysed during the current study are available from the corresponding author on reasonable request.

## Declarations

### Registration of Protocol

This systematic review was conducted following the Cochrane Handbook for Systematic Reviews of Interventions and Preferred Reporting Items for Systematic Reviews and Meta-Analyses 2020. The protocol was registered and can be accessed in Open science framework registries DOI :10.17605/OSF.IO/kcpvb.

## References

[1] Ogaard B, Rølla G, Arends J (1988). Orthodontic appliances and enamel demineralization. Part 1. Lesion development. Am J Orthod Dentofacial Orthop.

[2] Migliorati M, Isaia L, Cassaro A, Rivetti A, Silvestrini-Biavati F, Gastaldo L, Piccardo I, Dalessandri D, Silvestrini-Biavati A (2015). Efficacy of professional hygiene and prophylaxis on preventing plaque increase in orthodontic patients with multibracket appliances: a systematic review. Eur J Orthod.

[3] Ogaard B, Rølla G, Arends J, ten Cate JM (1988). Orthodontic appliances and enamel demineralization. Part 2. Prevention and treatment of lesions. Am J Orthod Dentofacial Orthop.

[4] Ogaard B (1989). Prevalence of white spot lesions in 19-year-olds: a study on untreated and orthodontically treated persons 5 years after treatment. Am J Orthod Dentofacial Orthop.

[5] Lundström F, Krasse B (1987). Streptococcus mutans and lactobacilli frequency in orthodontic patients; the effect of chlorhexidine treatments. Eur J Orthod.

[6] Lundström F, Krasse B (1987). Caries incidence in orthodontic patients with high levels of Streptococcus mutans. Eur J Orthod.

[7] Andrucioli MC, Nelson-Filho P, Matsumoto MA, Saraiva MC, Feres M, de Figueiredo LC, Martins LP (2012). Molecular detection of in-vivo microbial contamination of metallic orthodontic brackets by checkerboard DNA-DNA hybridization. Am J Orthod Dentofacial Orthop.

[8] Al-Melh MA, Bhardwaj RG, Pauline EM, Karched M (2020). Real-time polymerase chain reaction quantification of the salivary levels of caries-associated bacteria in patients with orthodontic fixed appliances. Clin Exp Dent Res.

[9] Rosvall MD, Fields HW, Ziuchkovski J, Rosenstiel SF, Johnston WM (2009). Attractiveness, acceptability, and value of orthodontic appliances. Am J Orthod Dentofacial Orthop.

[10] Rossini G, Parrini S, Castroflorio T, Deregibus A, Debernardi CL (2015). Periodontal health during clear aligners treatment: a systematic review. Eur J Orthod.

[11] Azeem M, Ul Hamid W (2017). Incidence of white spot lesions during orthodontic clear aligner therapy. J World Federation Orthodontists.

[12] Albhaisi Z, Al-Khateeb SN, Abu Alhaija ES (2020). Enamel demineralization during clear aligner orthodontic treatment compared with fixed appliance therapy, evaluated with quantitative light-induced fluorescence: a randomized clinical trial. Am J Orthod Dentofacial Orthop.

[13] Moshiri M, Eckhart JE, McShane P, German DS (2013). Consequences of poor oral hygiene during aligner therapy. J Clin Orthod.

[14] Yan D, Liu Y, Che X, Mi S, Jiao Y, Guo L, Li S (2021). Changes in the Microbiome of the Inner Surface of Clear Aligners after different usage periods. Curr Microbiol.

[15] Lu H, Tang H, Zhou T, Kang N (2018). Assessment of the periodontal health status in patients undergoing orthodontic treatment with fixed appliances and invisalign system: a meta-analysis. Med (Baltim).

[16] Shokeen B, Viloria E, Duong E, Rizvi M, Murillo G, Mullen J, Shi B, Dinis M, Li H, Tran NC, Lux R, Wu T (2022). The impact of fixed orthodontic appliances and clear aligners on the oral microbiome and the association with clinical parameters: a longitudinal comparative study. Am J Orthod Dentofacial Orthop.

[17] Chhibber A, Agarwal S, Yadav S, Kuo CL, Upadhyay M (2018). Which orthodontic appliance is best for oral hygiene? A randomized clinical trial. Am J Orthod Dentofacial Orthop.

[18] Pango Madariaga AC, Bucci R, Rongo R, Simeon V, D’Antò V, Valletta R. Impact of fixed orthodontic appliance and clear aligners on the periodontal health: a prospective clinical study. Dentistry J. 2020;8. 10.3390/dj8010004. 10.3390/dj8010004 PMC7175220 31906577

[19] Mummolo S, Nota A, Albani F, Marchetti E, Gatto R, Marzo G, Quinzi V, Tecco S. Salivary levels of Streptococcus mutans and lactobacilli and other salivary indices in patients wearing clear aligners versus fixed orthodontic appliances: an observational study. PLoS ONE. 2020;15. 10.1371/journal.pone.0228798. 10.1371/journal.pone.0228798 PMC7182227 32330172

[20] Wang Q, Ma JB, Wang B, Zhang X, Yin YL, Bai H (2019). Alterations of the oral microbiome in patients treated with the Invisalign system or with fixed appliances. Am J Orthod Dentofacial Orthop.

[21] Page MJ, McKenzie JE, Bossuyt PM, Boutron I, Hoffmann TC, Mulrow CD, Shamseer L, Tetzlaff JM, Akl EA, Brennan SE, Chou R, Glanville J, Grimshaw JM, Hróbjartsson A, Lalu MM, Li T, Loder EW, Mayo-Wilson E, McDonald S, McGuinness LA, Stewart LA, Thomas J, Tricco AC, Welch VA (2021). Whiting P and Moher D. The PRISMA 2020 statement: an updated guideline for reporting systematic reviews. BMJ.

[22] Karkhanechi M, Chow D, Sipkin J, Sherman D, Boylan RJ, Norman RG, Craig RG, Cisneros GJ (2013). Periodontal status of adult patients treated with fixed buccal appliances and removable aligners over one year of active orthodontic therapy. Angle Orthod.

[23] Sterne JAC, Savović J, Page MJ, Elbers RG, Blencowe NS, Boutron I, Cates CJ, Cheng HY, Corbett MS, Eldridge SM, Emberson JR, Hernán MA, Hopewell S, Hróbjartsson A, Junqueira DR, Jüni P, Kirkham JJ, Lasserson T, Li T, McAleenan A, Reeves BC, Shepperd S, Shrier I, Stewart LA, Tilling K, White IR, Whiting PF, Higgins JPT (2019). RoB 2: a revised tool for assessing risk of bias in randomised trials. BMJ.

[24] Sterne JA, Hernán MA, Reeves BC, Savović J, Berkman ND, Viswanathan M, Henry D, Altman DG, Ansari MT, Boutron I, Carpenter JR, Chan AW, Churchill R, Deeks JJ, Hróbjartsson A, Kirkham J, Jüni P, Loke YK, Pigott TD, Ramsay CR, Regidor D, Rothstein HR, Sandhu L, Santaguida PL, Schünemann HJ, Shea B, Shrier I, Tugwell P, Turner L, Valentine JC, Waddington H, Waters E, Wells GA, Whiting PF, Higgins JP (2016). ROBINS-I: a tool for assessing risk of bias in non-randomised studies of interventions. BMJ.

[25] Mummolo S, Tieri M, Nota A, Caruso S, Darvizeh A, Albani F, Gatto R, Marzo G, Marchetti E, Quinzi V, Tecco S (2020). Salivary concentrations of Streptococcus mutans and lactobacilli during an orthodontic treatment. An observational study comparing fixed and removable orthodontic appliances. Clin Experimental Dent Res.

[26] Abbate GM, Caria MP, Montanari P, Mannu C, Orrù G, Caprioglio A, Levrini L (2015). Periodontal health in teenagers treated with removable aligners and fixed orthodontic appliances. J Orofac Orthop.

[27] Levrini L, Abbate GM, Migliori F, Orrù G, Sauro S, Caprioglio A (2013). Assessment of the periodontal health status in patients undergoing orthodontic treatment with fixed or removable appliances. A microbiological and preliminary clinical study. Cumhuriyet Dent J.

[28] Levrini L, Mangano A, Montanari P, Margherini S, Caprioglio A, Abbate GM (2015). Periodontal health status in patients treated with the Invisalign® system and fixed orthodontic appliances: a 3 months clinical and microbiological evaluation. Eur J Dentistry.

[29] Buschang PH, Chastain D, Keylor CL, Crosby D, Julien KC (2019). Incidence of white spot lesions among patients treated with clear aligners and traditional braces. Angle Orthod.

[30] Dallel I, Ben Salem I, Merghni A, Bellalah W, Neffati F, Tobji S, Mastouri M, Ben Amor A (2020). Influence of orthodontic appliance type on salivary parameters during treatment. Angle Orthod.

[31] Alshatti H. “Comparison of White Spot Lesions among Clear Aligners, Self-Ligating Brackets and Conventional Brackets - A Randomized Controlled Clinical Trial” (2017). Master’s Theses. 1111. https://opencommons.uconn.edu/gs_theses/.

[32] Miethke RR, Vogt S (2005). A comparison of the periodontal health of patients during treatment with the Invisalign system and with fixed orthodontic appliances. J Orofac Orthop.

[33] Zamora-Martínez N, Paredes-Gallardo V, García-Sanz V, Gandía-Franco JL, Tarazona-Álvarez B. Comparative study of oral health-related quality of life (OHRQL) between different types of Orthodontic Treatment. Med (Kaunas). 2021;57. 10.3390/medicina5707068. 10.3390/medicina57070683 PMC8304849 34356964

[34] Oikonomou E, Foros P, Tagkli A, Rahiotis C, Eliades T, Koletsi D (2021). Impact of aligners and fixed appliances on oral health during Orthodontic Treatment: a systematic review and Meta-analysis. Oral Health Prev Dent.

[35] Bergamo AZN, Matsumoto MAN, Nascimento CD, Andrucioli MCD, Romano FL, Silva RAB, Silva LAB, Nelson-Filho P. Microbial species associated with dental caries found in saliva and in situ after use of self-ligating and conventional brackets. J Appl Oral Sci 201; 27:e20180426. doi: 10.1590/1678-7757-2018-0426. 10.1590/1678-7757-2018-0426 PMC6459231 30994775

[36] Bergamo AZ, Nelson-Filho P, Romano FL, da Silva RA, Saraiva MC, da Silva LA, Matsumoto MA (2016). Gingival crevicular fluid volume and periodontal parameters alterations after use of conventional and self-ligating brackets. J Orthod.

[37] Lombardo L, Ortan Y, Gorgun Ö, Panza C, Scuzzo G, Siciliani G (2013). Changes in the oral environment after placement of lingual and labial orthodontic appliances. Prog Orthod.

[38] Buck T, Pellegrini P, Sauerwein R, Leo MC, Covell DA, Maier T, Machida CA (2011). Elastomeric-ligated vs self-ligating appliances: a pilot study examining microbial colonization and white spot lesion formation after 1 year of orthodontic treatment. Orthod (Chic).

[39] Pandis N, Papaioannou W, Kontou E, Nakou M, Makou M, Eliades T (2010). Salivary Streptococcus mutans levels in patients with conventional and self-ligating brackets. Eur J Orthod.

[40] do Nascimento LE, de Souza MM, Azevedo AR, Maia LC (2014). Are self-ligating brackets related to less formation of Streptococcus mutans colonies? A systematic review. Dent Press J Orthod.

[41] Polat Ö, Gökçelik A, Arman A, Arhun N (2008). A comparison of white spot lesion formation between a self-ligating bracket and a conventional preadjusted straight wire bracket. World J Orthod.

[42] Nalçacı R, Özat Y, Çokakoğlu S, Türkkahraman H, Önal S, Kaya S (2014). Effect of bracket type on halitosis, periodontal status, and microbial colonization. Angle Orthod.

[43] Øgaard B, Larsson E, Henriksson T, Birkhed D, Bishara SE (2001). Effects of combined application of antimicrobial and fluoride varnishes in orthodontic patients. Am J Orthod Dentofacial Orthop.

[44] Jiang Q, Li J, Mei L, Du J, Levrini L, Abbate GM, Li H (2018). Periodontal health during orthodontic treatment with clear aligners and fixed appliances: a meta-analysis. J Am Dent Assoc.

[45] Higgins JPT, Thomas J, Chandler J, Cumpston M, Li T, Page MJ, Welch VA (2019). Cochrane Handbook for systematic reviews of interventions.

