# Supplementary material for: White spot lesions, plaque accumulation and salivary caries-associated bacteria in clear aligners compared to fixed orthodontic treatment. A systematic review and meta- analysis
Source: BMC Oral Health. 2023 Aug 27;23:599. doi: 10.1186/s12903-023-03257-8 (PMC10463770; doi:10.1186/s12903-023-03257-8)
Supplement: Supplementary file 1 — Supplementary Material 1 [file 12903_2023_3257_MOESM1_ESM.docx]

Online resource 1: List of excluded articles and the reasons for exclusion

| SL.NO | AUTHOR/YEAR | TITLE OF THE STUDY | REASON FOR EXCLUSION |
| --- | --- | --- | --- |
| 1 | Rego et al; 2010 [1] | Clinical and microbiological studies of children and adolescents receiving orthodontic treatment | Type of removable appliance not specified |
| 2 | Wang et al; 2019 [2] | Alterations of the oral microbiome in  patients treated with the Invisalign  system or with fixed appliances | Salivary cariogenic bacterial levels not investigated |
| 3 | Zancajo et al; 2020 [3] | Pain and Oral-Health-Related Quality of Life in  Orthodontic Patients During Initial Therapy with  Conventional, Low-Friction, and Lingual Brackets and  Aligners (Invisalign): A Prospective Clinical Study | Questionnaire- based study |
| 4 | Tuncay et al; 2013 [4] | Aligner Treatment  in the Teenage Patient | Comparator group (CF orthodontic appliance) not studied |
| 5 | Topaloglu et al; 2011 [5] | Effect of Orthodontic Appliances on Oral Microbiota—6 Month  Follow-up | Type of removable appliance not specified |
| 6 | Edward Lorilla Viloria | Characterization of the  Oral Microbiome in Orthodontic Patients | Type of fixed orthodontic appliance not specified |

REFERENCES:

1.Rego RO, Oliveira CA, dos Santos-Pinto A, Jordan SF, Zambon JJ, Cirelli JA and Haraszthy VI (2010) Clinical and microbiological studies of children and adolescents receiving orthodontic treatment. Am J Dent 23:317-23.

2.Wang Q, Ma JB, Wang B, Zhang X, Yin YL and Bai H (2019) Alterations of the oral microbiome in patients treated with the Invisalign system or with fixed appliances. Am J Orthod Dentofacial Orthop 156:633-640. doi: 10.1016/j.ajodo.2018.11.017

3.Antonio-Zancajo L, Montero J, Albaladejo A, Oteo-Calatayud MD and Alvarado-Lorenzo A (2020) Pain and Oral-Health-Related Quality of Life in Orthodontic Patients During Initial Therapy with Conventional, Low-Friction, and Lingual Brackets and Aligners (Invisalign): A Prospective Clinical Study. J Clin Med 9. doi: 10.3390/jcm9072088

4.Tuncay O, Bowman SJ, Amy B and Nicozisis J (2013) Aligner treatment in the teenage patient. Journal of clinical orthodontics : JCO 47:115-119; quiz 140.

5.Topaloglu-Ak A, Ertugrul F, Eden E, Ates M and Bulut H (2011) Effect of orthodontic appliances on oral microbiota--6 month follow-up. J Clin Pediatr Dent 35:433-6. doi: 10.17796/jcpd.35.4.61114412637mt661

Online resource 2: Summary statistics with Prediction interval

Random- effects model Method: REML

| Study | Plaque index | 95% C.I. | % Weight |
| --- | --- | --- | --- |
| Chhibber 2018 | -0.631 | -1.229 | 12.81 |
| Levrini 2013 | -1.138 | -2.048 | 12.07 |
| Meithke 2005 | -0.049 | -1.003 | 12.98 |
| Pango 2020 | -0.120 | -0.728 | 12.79 |
| Mummolo 2020 | -3.922 | -4.668 | 12.48 |
| Karkhanechi 2013 | -1.701 | -2.397 | 12.60 |
| Abate 2015 | -3.673 | -4.605 | 12.01 |
| Shokeen 2022 | -1.101 | -1.934 | 12.27 |
| Theta | -1.578 | -2.571 -0.585 |  |

Heterogeneity: tau ^2^= 1.9112, I^2^ = 93.85%, H^2^ = 16.26

95% prediction interval for theta: (-5.181, 2.025)

Test of homogeneity: Q= chi^2^ (7) = 102.76, Prob (Q) = 0.0000

Online resource 3: Meta-regression (REML model) with follow-up duration as continuous covariate (8 observations)

| Meta estimate | Coefficient | Standard error | z | P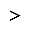 IzI | 95% C.I. |
| --- | --- | --- | --- | --- | --- |
| Duration | -0.0100 | 0.1122 | -0.09 | 0.929 | -0.22 , 0.20 |
| Constant | -1.4878 | 1.1748 | -1.27 | 0.205 | -3.79 , 0.81 |

Residual heterogeneity: Tau^2^= 2.256

I^2^= 94.57 %

H^2^= 18.43

R- squared= 0 %

Residual homogeneity: Q (res)= chi^2^(6)=102.71 (0.00)
